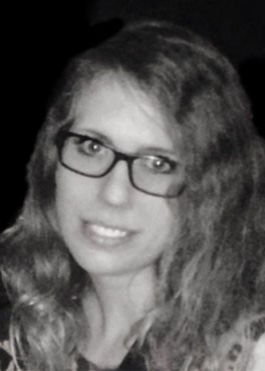# Chemical Biology Tools To Investigate Malaria Parasites

**DOI:** 10.1002/cbic.202000882

**Published:** 2021-03-31

**Authors:** Johannes Broichhagen, Nicole Kilian

**Affiliations:** ^1^ Leibniz-Forschungsinstitut für Molekulare Pharmakologie (FMP) Robert-Roessle-Strasse 10 13125 Berlin Germany; ^2^ Centre for Infectious Diseases Parasitology Heidelberg University Hospital Im Neuenheimer Feld 324 69120 Heidelberg Germany

**Keywords:** chemical biology, infectious diseases, malaria, parasites, *Plasmodium falciparum*, small-molecule probes

## Abstract

Parasitic diseases like malaria tropica have been shaping human evolution and history since the beginning of mankind. After infection, the response of the human host ranges from asymptomatic to severe and may culminate in death. Therefore, proper examination of the parasite's biology is pivotal to deciphering unique molecular, biochemical and cell biological processes, which in turn ensure the identification of treatment strategies, such as potent drug targets and vaccine candidates. However, implementing molecular biology methods for genetic manipulation proves to be difficult for many parasite model organisms. The development of fast and straightforward applicable alternatives, for instance small‐molecule probes from the field of chemical biology, is essential. In this review, we will recapitulate the highlights of previous molecular and chemical biology approaches that have already created insight and understanding of the malaria parasite *Plasmodium falciparum*. We discuss current developments from the field of chemical biology and explore how their application could advance research into this parasite in the future. We anticipate that the described approaches will help to close knowledge gaps in the biology of *P. falciparum* and we hope that researchers will be inspired to use these methods to gain knowledge – with the aim of ending this devastating disease.

## Graphical abstract

**Ghost hunting**: Malaria tropica has been haunting mankind for centuries, but investigating the biology of the causative agent, the protozoan parasite *P. falciparum*, remains challenging. Herein, we describe ready‐to‐use, non‐genetic, small molecular probes from the field of chemical biology that have been, or may be, used to interrogate the complex infection cycle, with an emphasis on infected red blood cells.

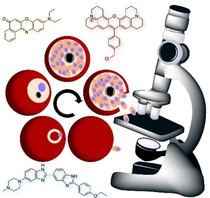

## 1 Investigating a Centuries‐Old Burden of Mankind

In classical Greek antiquity, a parasite (parasitos=παρασιτος) was an uninvited, but tolerated, guest during a meal.^[1]^ Around the time these dinners took place, the first written Egyptian records of what would later be defined as parasitic infections by modern medicine might have already been more than 1000 years old.^[2]^

Today, parasitic infections are still responsible for some of the most devastating diseases in the world, such as malaria tropica, caused by the protozoan parasite *Plasmodium falciparum* and responsible for 229 million clinical cases and approximately 400 000 deaths annually.[^2^, ^3^] Despite a coexistence of *P. falciparum* and humans for thousands of years, many aspects of parasite biology remain obscure to this day. One reason for this lack of knowledge is the challenging genetic manipulation of the parasite.^[4]^ While transient transfection is often the method of choice to investigate the behaviour and localization of distinct proteins in mammalian cell lines, stable transfections are usually needed to investigate *P. falciparum*.[^5^, ^6^] Various methods for genetic manipulation have been successfully established over the years and have already created immense insights into the biology of parasites (Table 1).[^6^, ^7^, ^8^, ^9^, ^10^] However, implementation and verification of many of these important methods can be time consuming, which is disadvantageous when multiple transgenic strains need to be generated and examined. Therefore, applicable alternatives that are straightforward to use and successfully deliver immediate results are in high demand to achieve a faster and better understanding of parasite biology.

**Table 1:** Selection of milestones in the genetic manipulation of *P. falciparum* blood stages.

Year	Method	Refs.
1993	Transient transfection	[7]
1995	Stable transfection	[8]
1996	Gene‐targeted/HR‐based	[177]
2006	Bxb1 integrase‐mediated site‐specific integration	[178]
2014	CRISPR/Cas9	[179–181]
2017	Selection‐linked integration (SLI)	[10]

## 2 The Biology of Plasmodium falciparum

**Figure 1:**
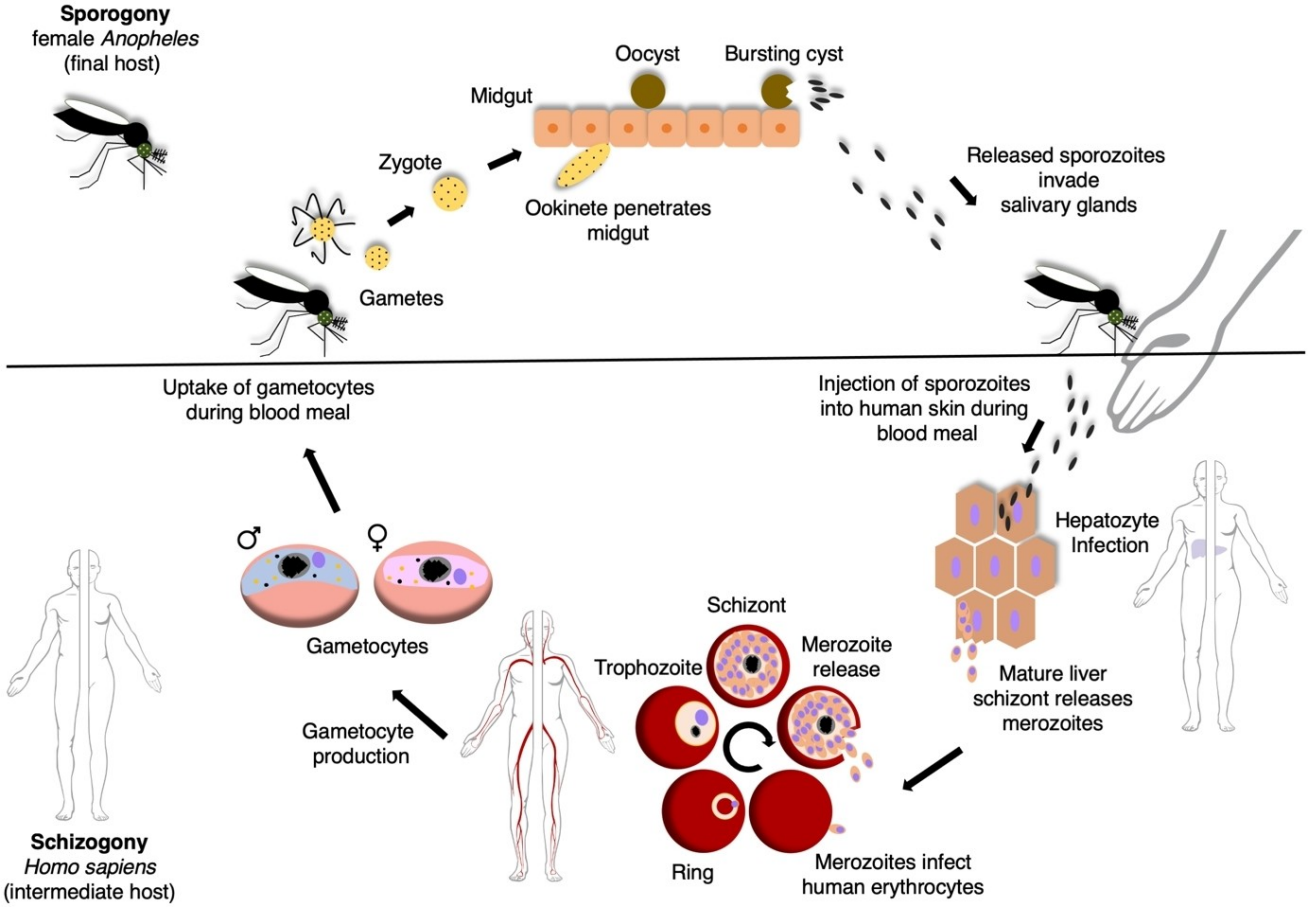
Life cycle of human‐pathogenic *Plasmodium* parasites. The asexual reproduction (schizogony, lower panel) takes place in the human intermediate host, while the sexual replication (sporogony, upper panel) occurs in the final host. During their life cycle, *Plasmodium* parasites are able to infect several cell types of the human host such as hepatocytes (liver schizogony) and RBCs (erythrocytic schizogony). Sexual precursor cells, so‐called gametocytes, are ingested by a female *Anopheles* mosquito during the blood meal and mate within the midgut after their maturation into gametes. The newly formed zygote further matures into an ookinete, which migrates across the midgut epithelial cells. After settling beneath the epithelial cells, the ookinete rounds up to form a sporozoite‐producing oocyst. The produced sporozoites are then inoculated into the human host during the mosquitoes’ next blood meal. Modified with permission from ref. [167]. Copyright: 2020, Wiley. Image credit for pictograms: Human Protein Atlas.^[168]^

*P. falciparum* is a parasite with a complex biology. The parasite requires a human intermediate host and a mosquito of the genus *Anopheles* as final host to execute its life cycle (Figure 1).[^11^, ^12^] Throughout its life cycle, *P. falciparum* must infect and inhabit various cell types to ensure life cycle progression and production of healthy progeny.[^11^, ^12^]

The infection of the human intermediate host begins with the blood meal of the female *Anopheles* mosquito. Sporozoites are inoculated in the skin during the mosquito bite via the secretion of the mosquito‘s anticoagulant saliva. The sporozoites must then reach a blood vessel that carries them to the liver sinusoids where they leave the bloodstream and infect hepatocytes. Afterwards, the sporozoites develop into liver schizonts that produce and release thousands of haploid merozoites into the bloodstream after rupture (liver schizogony). Of note, *P. vivax* and *P. ovale*, the causative agents of malaria tertiana, are two human‐pathogenic *Plasmodium* species which are able to form hypnozoites.^[13]^ These dormant developmental stages are able to persist within the liver and can cause a relapse of malaria even years after the initial infection.

The released liver merozoites immediately commence to infect red blood cells (RBCs). Within the RBC the parasite matures starting from the young ring via trophozoite to the schizont developmental stage (erythrocytic schizogony). During the ring stage the parasite is highly mobile but grows slowly while expressing proteins that will eventually be exported into the host cell.[^14^, ^15^] After about 24 h the parasite develops into a stationary, hemoglobin‐ingesting and fast‐growing trophozoite.^[15]^ Finally, the parasite enters the schizont stage during which it generates up to 32 daughter merozoites. These newly developed merozoites are released into the bloodstream after schizont rupture and invade new RBCs. The erythrocytic schizogony of the parasite is responsible for the pathophysiology of malaria. Merozoite egress releases a bevy of pyrogens from the destroyed host cells, causing the hallmark fevers.^[16]^

To complete the erythrocytic schizogony, an exceptional reorganization of the terminally differentiated, metabolically reduced and organelle‐deprived host RBC is necessary to ensure nutrient supply, life cycle progression and protection against the immune response of the human host. This exceptional reorganization culminates in the generation of the Maurer's clefts, an enigmatic secretory organelle the parasite establishes within the cytoplasm of the host RBC to conduct the secretion of essential proteins for the inauguration of host‐parasite interaction.[^17^, ^18^] Successful genesis of the Maurer's clefts enables the secretion of PfEMP1 (*P. falciparum* erythrocyte membrane protein 1) to the RBC surface. PfEMP1 is a crucial virulence factor encoded by a family of roughly 60 *var* genes that are distributed across subtelomeres and internal regions of the parasite chromosomes.[^19^, ^20^] To achieve antigenic variation of the infected RBCs’ surface, the parasite regularly exchanges the expressed *var* gene. PfEMP1 further serves as an adhesive molecule that binds to an array of human receptors once it is presented on RBC plasma membrane protrusions (knobs).[^17^, ^21^] Cytoadherence and sequestration of RBCs infected with mature developmental stages of *P. falciparum* (trophozoite and schizont) are essential to avoid the spleen passage where deformed, senescent and infected RBCs are removed from blood circulation.^[21]^ Therefore, only young ring stages and mature stage V gametocytes (sexual developmental stages), which are supposed to be transmitted to the *Anopheles* mosquito (see below), are detectable in the peripheral blood of the patients while the remaining asexual and sexual developmental stages sequester.^[22]^ The increased dwelling time of infected RBCs within inner organs can lead to severe complications that might culminate in patient death. Hemoglobinopathies, such as sickle cell anaemia and thalassemia, have evolved as protective responses against these severe complications.^[21]^ Within these RBCs *P. falciparum* cannot establish properly functioning Maurer's clefts, which leads to a delay in protein secretion and a decreased amount of secreted proteins that are vital to establish a successful cytoadherence.[^17^, ^21^, ^23^]

During the erythrocytic schizogony some parasites eventually begin to differentiate into sexual developmental stages (gametocytes, sexual precursor cells: female macrogametocytes and male microgametocytes). The gametocytes are ingested by a female *Anopheles* mosquito during the blood meal and commence the sporogony.[^24^, ^25^] Gametocytes mature into gametes within the midgut of the mosquito and mate to form a zygote. This zygote develops into an ookinete that penetrates the midgut epithelial cells and develops into a sporozoite‐producing oocyst. The sporozoites are released after oocyst rupture and invade the salivary glands of the mosquito. These sporozoites can then again be inoculated into the human intermediate host during the next blood meal of the mosquito.

## 3 Chemical Biology Tools in Plasmodium falciparum Research

Many aspects of parasite biology still remain shrouded in mystery today. The erythrocytic schizogony is mainly responsible for the symptom manifestation. A fast and successful investigation of this important part of the parasite's life cycle is therefore crucial to identify novel vaccine and drug targets in addition to the transmission‐blocking drugs and vaccines that interfere with gametocyte transmission, sporozoite transmission or aim at the merozoites.[^26^, ^27^, ^28^, ^29^, ^30^] Primaquine, for example, is the only WHO‐recommended transmission‐blocking drug to date.^[31]^ However, toxicity issues in G6PD (glucose‐6‐phosphate dehydrogenase) deficient individuals hampers an extensive use of this drug.^[31]^

Research tools developed by the field of chemical biology can support malaria researchers in their quest to demystify the parasite. Chemical biology uses the power of organic chemistry to design and synthesize small molecules that can be applied in biological systems. There are many applications in labelling and visualizing biomolecules and we aim to highlight established approaches that have not been used frequently in parasitology research to date. Our focus will be chemical biology probes that can be applied directly to the native system without the need for genetic editing. To that extent, we will give an overview of recent developments in the field, explain the tools already in use and assess where in parasitology research novel probes could be applied. We discuss the implementation of small‐molecule sensors with an emphasis on the intricate system of *P. falciparum* infectivity on RBCs and we will provide an outlook of which chemical biology probes are next in line for the interrogation of the complex interplay between the parasite and its host. We focus on fluorescent imaging, a technique at the forefront of interrogating biology in its live and native context. By pointing towards future directions, we hope to inspire parasitology researchers to equip their repertoire with ready‐to‐use small‐molecule reporters.

### 3.1 Microscopy of P. falciparum‐infected RBCs

**Figure 2:**
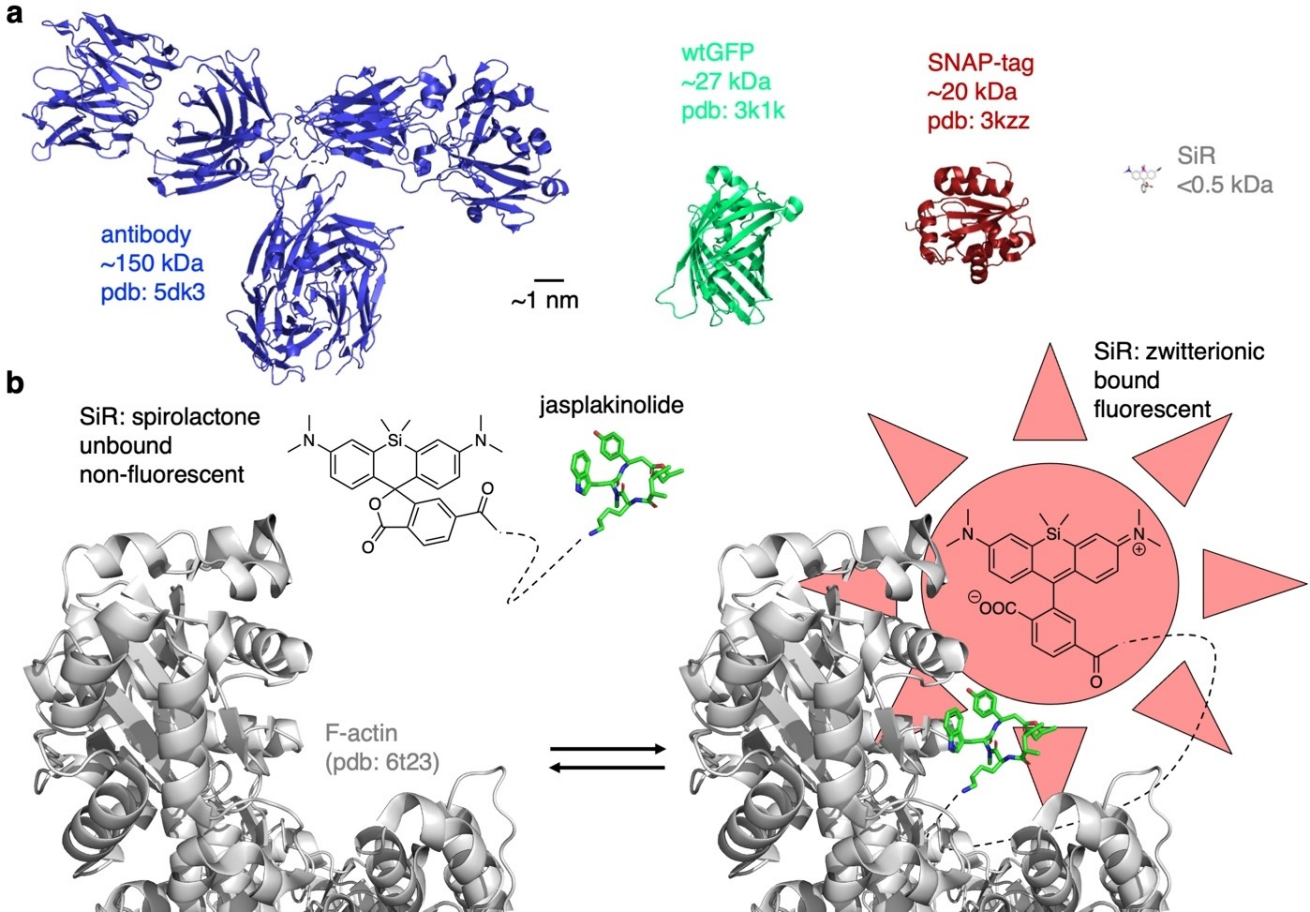
Labelling molecules and fluorogenic dyes. a) Relative sizes of an antibody (PDB ID: 5DK3^[169]^), green fluorescent protein (PDB ID: 3K1K^[170]^), a SNAP‐tag (PDB ID: 3kzz) and Silicon Rhodamine (SiR) dye (modelled) are shown for comparison. b) The logic of fluorogenicity. A molecule can be in two isomeric states, a non‐fluorescent form and a fluorescent one, where only the latter shows emission upon application of excitation wavelength. The fluorogenic molecule silicon rhodamine is shown linked to the actin binder jasplakinolide. It exists in its unbound, nonfluorescent and closed spirolactone form, which is converted to an open, zwitterionic form that turns on fluorescence upon binding to actin (PDB ID: 6T23^[171]^).

Live cell imaging of *P. falciparum*‐infected RBCs is particularly important to visualize protein trafficking, movement of parasite or host cell membranes and uptake of antimalarial drugs. The technique of choice in biological research is fluorescent microscopy, which relies on the introduction of fluorophores specifically localizing to a target of interest. Fluorescence is widely used in widefield and confocal imaging, yet resolution is limited to half the wavelength used for imaging (Abbe's law), which makes the detection of smaller structures difficult. This is especially the case for RBCs, which have a concave shape of only 8 μm in diameter and are the smallest cells found in the human body (e. g., skin cell ∼30 μm; neuron body ∼100 μm). One possibility to observe these fine structures is the use of electron microscopy, which yields resolution down to the one‐digit nanometre. However, this technique requires careful sample fixation and preparation, and is therefore not amenable in live cells. Still, it has been applied in the field of malaria research frequently. Hallmark studies have shown, for example, the development of the parasite, RBC reorganization, cytoadherence and the shape changes of gametocytes.[^21^, ^32^, ^33^] Advances in the optical field have led to an available system that circumvents Abbe's law by different techniques. As such, stimulated emission depletion (STED) and photo‐activated localization microscopy/stochastic optical reconstruction microscopy (PALM/STORM) have emerged as nanoscopic methods that allow detailed image acquisition down to the tens of nanometres.[^34^, ^35^, ^36^] In order to introduce fluorophores, the most popular approach uses genetic editing, more precisely cloning and transfection of an expression vector encoding for a fluorescent protein (FP) fusion, which can be targeted to cells to be visualized^[37]^ (Figure 2a). Coming with a size of ∼27 kDa and in a plethora of different photophysical properties, such as colour and brightness, the impact of these proteins cannot be understated (see www.fpbase.org for reference). In order to further boost photophysical properties and stability, self‐labelling protein tags have been used in recent years, with the SNAP‐ and Halo‐tag being the most popular.[^37^, ^38^] While they can be equipped with small organic fluorophores that offer unmatched brightness and stability, they still rely on genetic engineering of the biological system and exhibit usual sizes for globular protein domains (Halo: ∼30 kDa; SNAP: ∼20 kDa). As genetic engineering proved not as straightforward for parasite cell systems compared to mammalian (immortalized) cell systems,^[5]^ probes from the field of chemical biology are in demand that can be readily applied. Therefore, noninvasive investigation of the native system with fluorescent probes is crucial to ensure that the experimental approach mimics the *in vivo* situation accurately. As such, the use of fluorescently‐labelled antibodies generally enables the investigation of native systems. However, their size (∼150 kDa) and nonstoichiometric fusion to fluorophores result in trade‐offs for precise imaging (Figure 2a). This mainly results in fluorophores being far away from its target, thereby limiting the targets localization in super‐resolution approaches. Small molecular (∼0.5 kDa) smart dyes (Figure 2a), on the other hand, show fluorogenicity, meaning they exist in two states where they are able to toggle in between^[39]^ an unbound, non‐fluorescent form, and a target bound fluorescent form (Figure 2b, c). The specificity is accomplished by chemical fusion to a selective and strong target binder. This not only ensures specific tracing but also offers the possibility to perform fast “no‐wash” protocols, as the unbound form remains optically silent. As these probes exhibit intrinsic pharmacology, they furthermore bear the potential for diagnostic and therapeutic application.

### 3.2 Targeting organelles of parasite origin

The distinguished niches for the erythrocytic schizogony of *P. falciparum* are the RBCs of the human host. RBCs are unique cells with a typical lifespan of 120 days. During this time the RBCs constantly circulate through the bloodstream and squeeze through arteries, veins and small capillaries to inaugurate tissue respiration. To execute this important task, RBCs hold large numbers of haemoglobin and are deprived of intracellular organelles and consist of a distinct biconcave shape which is maintained by a well‐defined membrane skeleton. This membrane skeleton is a pseudohexagonal meshwork made of spectrin, actin, protein 4.1R, ankyrin and actin‐associated proteins that laminates the inner membrane surface and attaches to the overlying lipid bilayer via band 3‐containing multiprotein complexes at the ankyrin‐ and actin‐binding ends of spectrin.^[40]^

Blood stages of *P. falciparum* that inhabit the RBCs of the human host consist of several classical organelles such as the nucleus, endoplasmic reticulum (ER), mitochondria and Golgi apparatus that can also be found in mammalian cells. To ensure that organelle genesis and behaviour as well as general remodelling activities remain unaffected, fluorescent probes must warrant minimal interference while maintaining excellent stability over the course of imaging time.

#### 3.2.1 Nucleus

**Figure 3:**
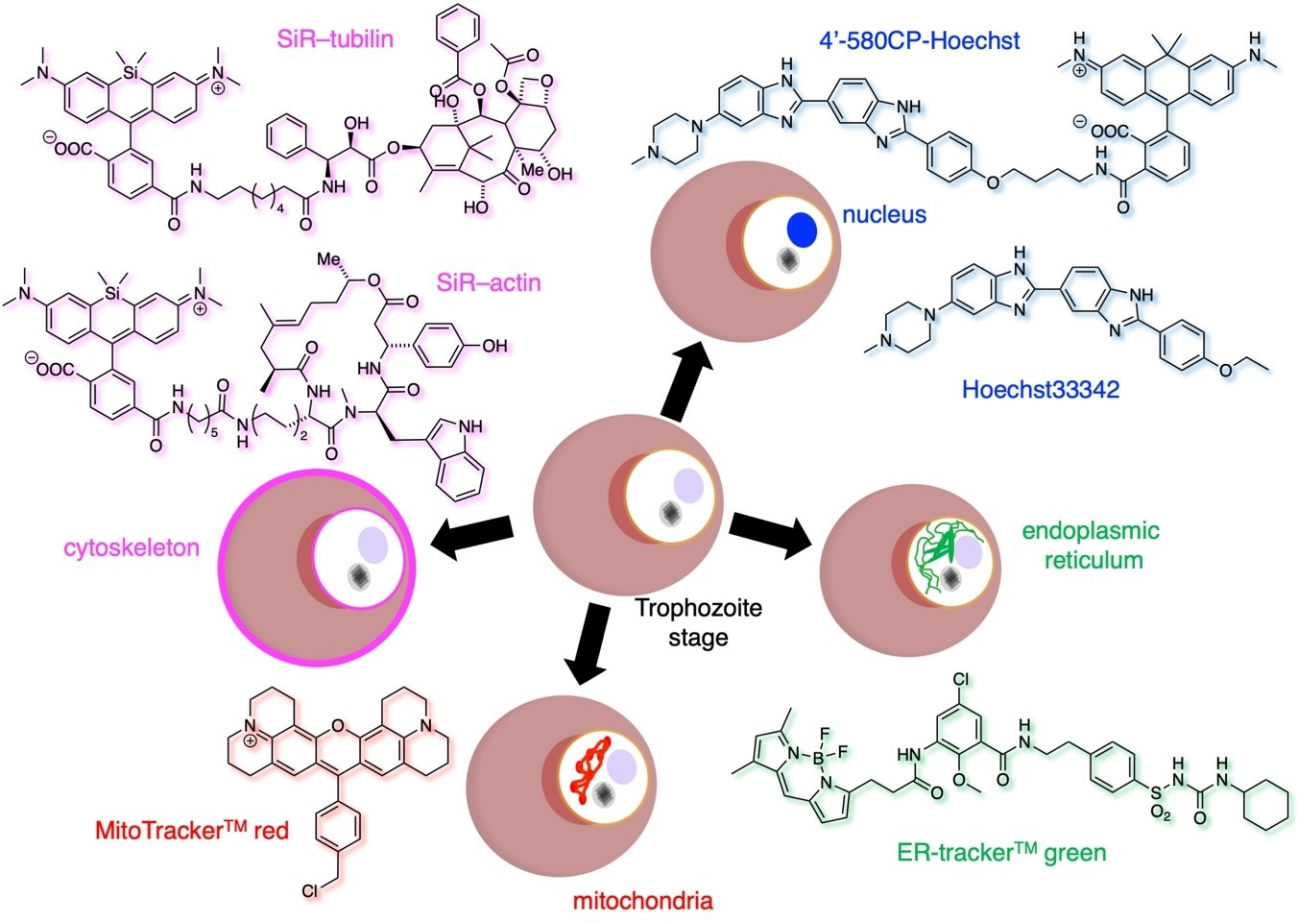
Organellar targeting of *P. falciparum*. Nuclear staining by Hoechst33342 can be achieved through DNA binding. When equipped with fluorophores with different linkers, colours can be tuned and UV light avoided, as exemplified 4′‐580CP‐Hoechst. The commercially available ER‐Tracker green and MitoTracker Red are used to visualize the ER and mitochondria, respectively. SiR has been used to target both the actin (SiR‐actin, *cf*. Figure 2) and tubulin (SiR‐tubulin) networks by linkage to jasplakinolide and paclitaxel congeners, respectively.

**Figure 4:**
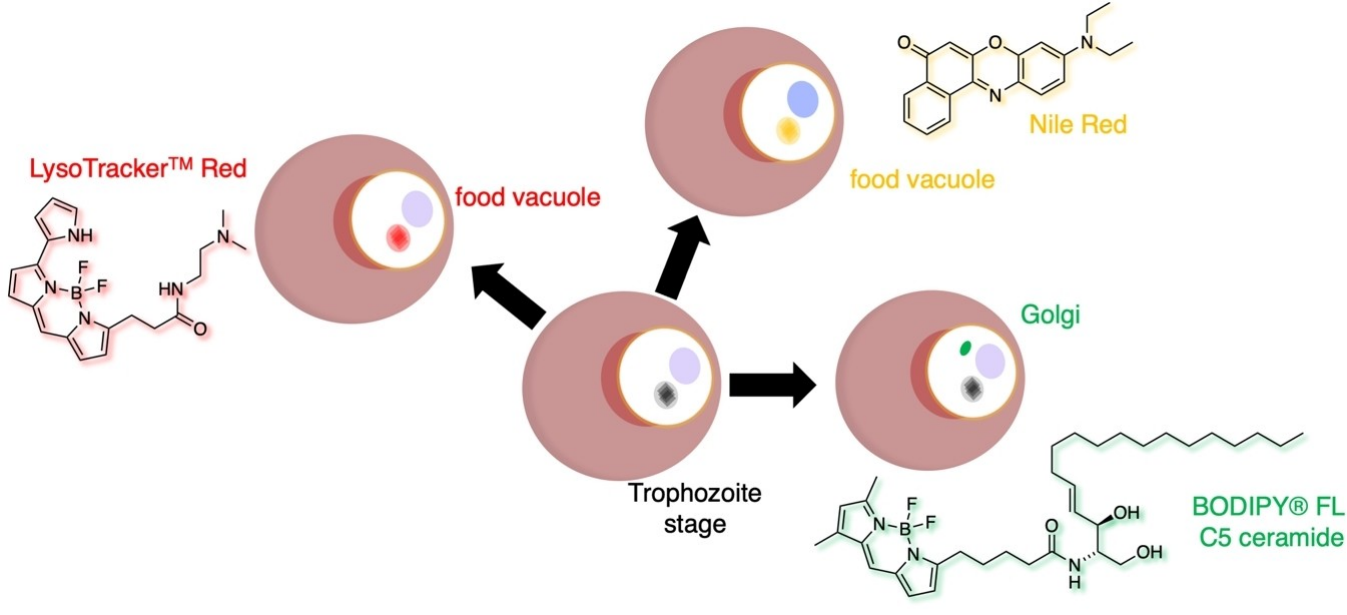
More organellar targeting of *P. falciparum*. Food vacuolar staining was performed with LysoTracker and Nile Red. The Golgi apparatus is amenable to being visualized with ceramide‐linked fluorophores from the BODIPY family.

Typically, Hoechst dyes are applied for live‐cell imaging of DNA (Figure 4a, below) and represent the standard method to investigate the nucleus of the different developmental stages of *P. falciparum* or to determine the presence of the parasite within different host cells such as RBCs or hepatocytes.[^41^, ^42^, ^43^, ^44^] Interestingly, Boehme and co‐workers used Hoechst33258‐cesium chloride (CsCl) ultracentrifugation to separate the AT‐rich DNA of the avian malaria parasite *Plasmodium gallinaceum* from the other DNA of the infected nucleated chicken RBCs.^[45]^ Hoechst binds to the minor groves of the DNA where it fluoresces brightly, which allows a straightforward visualization. However, the use of toxic UV light for fluorescent imaging can affect the health of *P. falciparum*. Therefore, Hoechst has been chemically fused to fluorophores (e. g., TMR to yield hoeTMR^[46]^) that absorb in the visible part of the spectrum. For instance, 4′‐580CP‐Hoechst^[47]^ and SiR‐Hoechst (also known as SiR‐DNA)^[48]^ are red and far‐red DNA stains, respectively, that are STED compatible and a “healthier” alternative (Figure 3). We recently reported a photocaged DNA stain termed pcHoechst, which was used to label sub‐compartmental regions of nuclear DNA in live cells and allowed *in vivo* DNA tracking in the zebrafish (*Danio rerio*) while ensuring animal health.^[49]^ These modern DNA targeting stains offer great potential in parasite research but have hitherto not been used to examine *P. falciparum*.

SYBR green, a DNA‐intercalating asymmetrical cyanine dye that binds preferably to the G and C base pair of double‐stranded DNA, is usually applied to determine the presence of *P. falciparum* when antimalarial drugs are investigated^[50]^ or to detect infected RBCs via flow cytometry.[^50^, ^51^, ^52^] However, Guy and co‐workers also applied the dye to simplify the microscopy‐based diagnosis of *P. falciparum*.^[53]^

#### 3.2.2 Endoplasmic reticulum and mitochondria

Other standard organelle targeting dyes, such as the ER‐ and MitoTracker, have been used to visualize the endoplasmic reticulum and mitochondria, respectively. While the ER‐Tracker consists of a sulfonylurea fused fluorophore (Figure 3), the MitoTracker usually relies on positively charged molecules that accumulate in the mitochondria (e. g., rhodamines, alkyltriphenyl phosphonium ions; Figure 3), due to its negative membrane potential. Recently, membrane tension sensing dyes have been equipped with these targeting moieties to measure biophysical membrane properties in different compartments.^[40]^ Both MitoTracker and ER‐Tracker have been successfully applied to investigate *P. falciparum*.[^54^, ^55^] In detail, Yeoman and co‐workers used the ER‐Tracker in their study to determine the localization of the transmembrane *P. falciparum* glideosome‐associated protein 45 (PfGAP45) before recruitment to its final destination in the inner membrane complex of the parasite.^[54]^ In a study published by Günther and co‐workers in 2007, MitoTracker was also used to identify the localization of LplA2 (lipoic acid protein ligase A1).^[55]^ They determined that LpIA2 is present in the mitochondrion and apicoplast of the parasite during the development of *P. falciparum* within the RBCs. Both ER‐Tracker and MitoTracker have also been applied to investigate the sexual developmental stages of *P. falciparum*. The MitoTracker Red CM‐H_2_XRos was further used as viability marker in two anti‐gametocyte HTS (high throughput screening) confocal fluorescence microscopy‐based assays to measure the anti‐gametocidal activity by assessing the number and viability of gametocytes in the investigated assay well.^[56]^ Olivieri and co‐workers applied the ER‐Tracker to investigate anomalous structures within *P. falciparum* as an effect of a knock‐out of *pfg27*, encoding for an RNA‐binding phosphoprotein essential for gametocyte production.^[57]^

#### 3.2.3 Cytoskeleton

Actin is an important cytoskeletal component for maintenance and function of parasite and host cell infrastructure.[^21^, ^23^, ^58^] To visualize F‐actin, the STED compatible dye silicon rhodamine (SiR) was chemically fused to des‐bromo‐des‐methyl‐jasplakinolide, an actin binding moiety, to obtain SiR‐actin^[59]^ (Figure 3). The initial report of SiR‐actin revealed successful staining of actin in uninfected RBCs and even allowed a 3D reconstruction of the RBCs based on the acquired confocal images.^[59]^ Both excitation and emission of SiR in the far‐red spectrum are an advantageous feature for the examination of RBCs because the whole blood absorption is minimized and the fluorogenicity increases up to ∼100‐fold upon actin binding.^[59]^ Of note, in a recently published study from the fall of 2020, Trisnadi and Barillas‐Murya fed the nontoxic SiR‐actin to *Anopheles* mosquitoes to visualize the reorganization of epithelial actin after the invasion of the ookinete in a live cell imaging approach.^[60]^ SiR‐tubulin (with a SiR‐fused paclitaxel group for targeting; Figure 3) is another probe to simplify the investigation of another important part of the parasite's cytoskeleton and has already been applied to investigate sporozoites of the *Plasmodium* species *P. berghei* and *P. yoelii*.[^61^, ^62^] It should be noted that SiR‐actin and SiR‐tubulin are stabilizing the cytoskeletal network by inhibiting the polymerization and therefore careful interpretation is warranted when interpreting actin and tubulin growth and shrinking.

#### 3.2.4 Golgi and lysosomes

The Golgi apparatus is a secretory organelle responsible for post‐translational protein modification, protein secretion, lysosome formation and lipid transport. Commercially available probes to label this important organelle are usually based on ceramide, a lipid comprising sphingosine and a fatty acid that targets the Golgi membrane.^[63]^ BODIPY®‐TR‐C5‐ceramide has for example been used to investigate the cavity, an invagination of the PVM that is visible during the erythrocytic schizogony of *P. falciparum* ^[15]^ (Figure 4). The probe, however, is notorious for labelling membranes of both parasite and host cell origin.

Probes that bear basic functionalities, such as morpholines and tertiary amines in general, are used to target the acidic compartments of lysosomes (Figure 4). As they become protonated, they are not able to leave this organelle and accumulate there over time. As such, LysoTracker accumulates in the acidic FV of the parasite,^[64]^ which has also been observed when Nile Red (Figure 4) was used as a lipophilic stain (*vide infra*).

#### 3.2.5 Membranes

**Figure 5:**
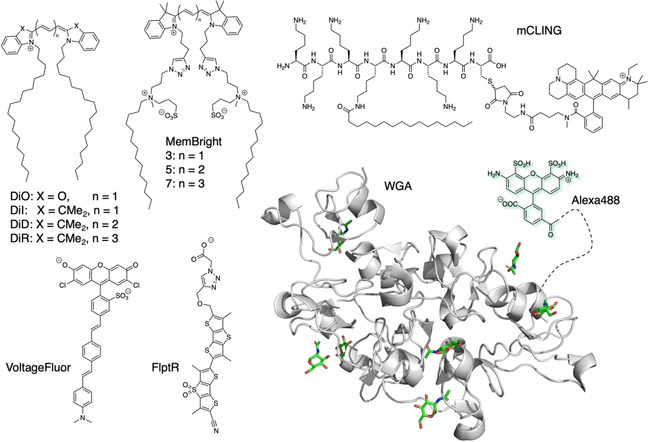
Lipid‐based probes. Membrane‐inserting probes DiO/DiI/DiD/DiR and MemBright versions are shown, the last of which forms micelles until insertion into the plasma membrane, enabling no‐wash protocols. mCLING is a cationic, palmitoylated peptide that is fused to ATTO647N, which attaches to the membrane and labels the endo/lysosomal network upon exposure. Membrane‐inserting dyes VoltageFluor and FliptR insert into the lipid bilayer and can sense changes in membrane potential and tension, respectively. Wheat germ agglutinin (WGA, PDB ID: 2UVO^[172]^) binds to sialic acid and *N*‐acetylglucosamine found on glycosylation patterns on proteins on the extracellular side. Fused to Alexa488, it fluoresces and is used to stain the cell's membranous system.

Lipids serve many functions, from building the lipid bilayer for cell membranes and therefore compartment generation, to protein modification and signal transduction. The molecular composition of membranes results in different biophysical properties. In addition, membrane curvature has biological impacts itself, for instance in endo‐ and exocytosis. Given the general importance and extensive remodelling of parasite membranes after and during infection, observing membrane fluxes can be exploited as a way to determine the infective cycle.^[15]^ Spatiotemporal precise observation of the host and parasite may provide insights into the infection process and the underlying biology. Membrane stains that insert into the lipophilic part of the bilayer are well described with new probes still emerging, allowing the tracking of lipid environments and performing super resolution microscopy.[^65^, ^66^] Examples include the colourful palette of DiO/DiI/DiD/DiR (Dioctadecyl) probes, which are cyanine dyes (Cy2/Cy3/Cy5/Cy7) linked to two C18 alkyl chains that insert into membranes,^[67]^ or WGA (wheat germ agglutinin) lectin, a carbohydrate binding moiety with high affinity for sialic acid and *N*‐acetylglucosamine,^[67]^ which are glycosylation patterns on the extracellular part of transmembrane proteins (Figure 5). When linked to a fluorophore like Alexa488, WGA brightly stains mammalian cell membranes. More recently, MemBright dyes were developed that link long alkyl chains to a cyanine fluorophore (e. g., Cy3/Cy5/Cy7), which is separated by charged ammonium and sulfonate groups. This amphiphilic dye forms non‐fluorescent micelles that insert into the membrane upon exposure. This in turn breaks the aggregates and exhibits a turn‐on^[66]^ (Figure 5). Lastly, mCLING (membrane‐binding fluorophore‐cysteine‐lysine‐palmitoyl group) has been developed that consists of an ATTO647 N fluorophore linked to a palmitoylated peptide (Figure 5) to stain the lysosomal system after membrane binding without washing and insensitive to fixation.^[65]^ Given the importance of membranes, their localization and dynamics, novel membrane highlighting probes can be used in live cell imaging approaches. Of particular interest are membrane probes that allow super‐resolution snapshots, such as mCLING and MemBright.

Another feature of the lipid bilayer is the separation of ions, which results in the building of a membrane potential due to the contribution of ion channels and pumps. Measuring this potential and the quick changes evoked by opening and closing of ion channels can be physically achieved by the patch clamp technique.^[68]^ While powerful, this technique is limited by its low throughput and ideally, fluorescent sensors would be advantageous. Indeed, fluorescent dyes that incorporate into the membrane and respond quickly to changes in membrane potential have been reported.[^69^, ^70^, ^71^] VoltageFluor (Figure 5), for instance, is able to give resolution to action potential firing in neurons, thereby displaying their fast and accurate nature to translate ion flow to an optical signal. This approach has been expanded to more dye scaffolds and colours, serving as a ready‐to‐use toolset for the measurement of ion flow that is evoked by synaptic transmission.^[71]^ From a more biophysical angle, the Matile and Roux groups have developed fluorescent dyes, termed FliptR (Figure 5), that are able to sense changes in membrane tension by fluorescence lifetime microscopy (FLIM) due to rotational restraints induced by their environment and are also applicable to super resolution techniques.[^72^, ^73^, ^74^] For instance, different lifetimes were recorded between mitochondria and the tubular ER, and even more, multiplexed microscopy revealed different membrane stiffness for the nuclear envelope, the ER, and lysosomes. As such, measurements in COS7 cells during mitochondrial constrictions that precede fission showed that membrane tension increases during the compression process.^[73]^ Given that *P. falciparum* remodels the RBCs of its host extensively by bringing and generating organellar structures (e. g., knobs on the RBCs surface, Maurer's clefts, parasitophorous vacuole membrane (PVM), tubovesicular network (TVN)), the usage of such small molecules would be an attractive method to gain insights into membrane remodelling and biophysical parameters of the parasite's actions. Indeed, studies on measuring changes in membrane potentials of the isolated trophozoite stages of the parasite were performed using radio‐labelled compounds and the voltage indicator bis‐oxonol in response to, for instance, proton pump inhibitors, glucose deprivation and potassium channel blockers.^[75]^ Much can be learned by further examining the membrane characteristics of *P. falciparum* isolated from or residing in the host in different stages and in combination with ion fluxes of the infected RBC.

#### 3.2.6 Other organelles

As mentioned above, *P. falciparum* remodels RBCs and forms organelles not found in healthy mammalian cells. One organelle is the apicoplast, a unique chloroplast‐like organelle that *P. falciparum* has acquired *via* secondary endocytosis. The apicoplast is responsible for an isoprenoid precursor and fatty acid synthesis and contributes to the heme biosynthetic pathway.[^76^, ^77^] The apicoplast's uniqueness destines the organelle as a drug target with high potential. However, the majority of studies still examine the apicoplast *via* immunofluorescence analyses or transgenic *P. falciparum* strains. The above‐mentioned pcHoechst is a probe with great potential to label the DNA and indirectly investigate the behaviour of the apicoplast.^[49]^ Similarly, micronemes, rhoptries and dense granules are secretory organelles involved in RBC infection and reorganization.[^78^, ^79^] These important organelles are, to the best of our knowledge, currently only investigated by immunofluorescence analyses or fluorescently tagged proteins, although with super‐resolution quality by structured illumination (SIM).^[80]^ This can be regarded as an opportunity where Chemical Biology probes might help to broaden the knowledge about these important organelles without the need for fixation and immunofluorescence imaging.

Another essential organelle of *P. falciparum* is the acidic food vacuole (FV) necessary for haemoglobin degradation, haem polymerization, amino acid transport, detoxification of oxygen radicals, accumulation of drugs, maintenance of acidification and generation of free iron.^[81]^ Due to the lack of morphological evidence for an endosome‐lysosome system within the parasite, the proteases within the FV might allow the organelle to act as a lysosome‐like compartment. A study by Hayward et al. from 2006 has already applied several dextran‐linked pH‐sensitive fluorescent dyes to investigate the pH of the organelle in chloroquine‐sensitive and chloroquine‐resistant strains of *P. falciparum*.^[82]^ Klonis and co‐workers applied LysoSensor Blue to confirm the acidity of the FV in addition to comparing the pH of the organelle with that of the ER and cytosomal vesicles in a manuscript published in 2007,^[83]^ alongside the use of LysoTracker Red (Figure 4).

In 2004, Palacpac and co‐workers applied the hydrophobic probe Nile Red to visualize neutral lipid bodies (NLB) in *P. falciparum*‐parasitized erythrocytes and stated the observed lipid structures are secretory pathway‐associated and eventually accumulate in the parasitophorous vacuole (PV), a membrane that surrounds the parasite and originates from the RBC‐infection.^[84]^ In the same year the Tilley laboratory also applied Nile Red to investigate *P. falciparum* and observed an association of lipid bodies with the FV rather than the PV^[85]^ (Figure 4). They further discovered the lipid components of these structures, or possibly their precursors, are connected to haem detoxification in the FV.

### 3.3 Reporters and sensors

**Figure 6:**
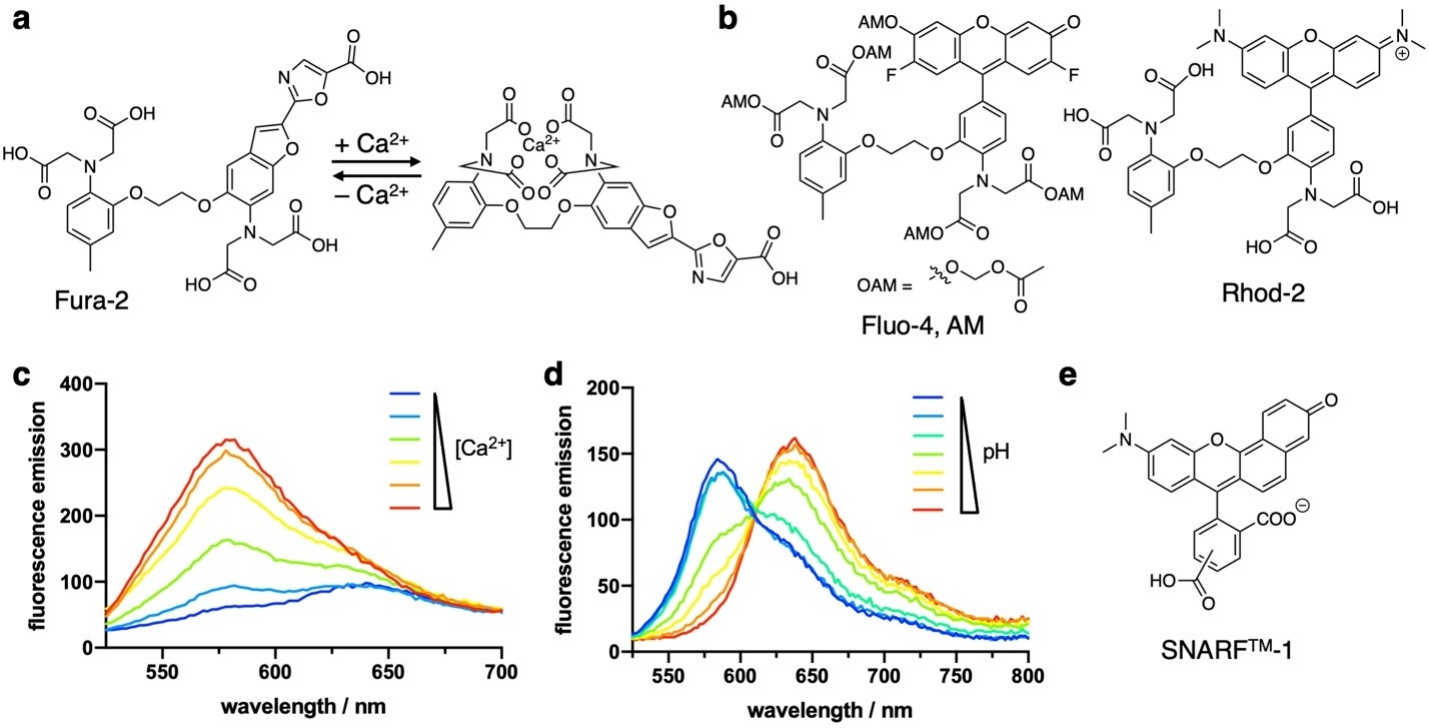
Probes for sensing ion fluctuations. a) Reporter Fura‐2 binds Ca^2+^, thereby changing its excitation properties for live‐cell Ca^2+^ imaging. b) Fluo‐4, AM and Rhod‐2 are shown for live‐cell Ca^2+^ imaging. Acetoxymethoxy (AM) esters can be used to mask the charged carboxylates for intracellular delivery, where esterase liberated the Ca^2+^ sensing unit (*cf*. Fluo‐4). c) Example of intensiometric behaviour. Upon increasing Ca^2+^ concentration, an increase in fluorescence can be detected by a single emission band. d) Example for a ratiometric behaviour. Two emission bands change diametrically upon pH changes. e) SNARF‐1 is a ratiometric pH sensor.

Small‐molecule reporters and sensors are used to image and ideally quantify intracellular analyte concentrations and their change over time. For this reason, they have to exhibit specificity towards their binding target with a dynamic response in the physiological concentration range. Small‐molecule calcium (Ca^2+^) dyes serve as a prominent example. Developed in the 1980s by Tsien, they consist of a BAPTA group (1,2‐bis(*o*‐aminophenoxy)ethane‐*N*,*N*,*N*’,*N*’‐tetraacetic acid) that selectively binds free Ca^2+^ ions, fused to a benzofuran oxazole, yielding the well‐known Fura‐2 dye^[86]^ (Figure 6a), which has been applied in *P. falciparum* research multiple times.[^87^, ^88^] As such, it was observed that reduction of the extracellular Ca^2+^ concentration leads to a Ca^2+^ depletion in the parasitophorous vacuole, in turn affecting the maturation process of *P. falciparum* within its host.^[88]^ While ratiometric Ca^2+^ sensing of Fura‐2 by two excitation colours is an advantage, the use of UV light is a drawback. Therefore, the cell‐permeant and green emitting, yet intensiometric, dye Fluo‐4 AM has been used previously to study the behaviour of Ca^2+^ in the blood stages of *P. falciparum*.[^89^, ^90^, ^91^] In a manuscript published in 2014, Friedrich and co‐workers applied Fluo‐4 AM (Figure 6b) to investigate the transport rate of PfMDR1 (*P. falciparum* multidrug resistance transporter 1), a transporter involved in chloroquine resistance of the parasite.^[92]^ A red‐shifting spectral characteristic Ca^2+^ dye, Rhod‐2 AM (red emitting; Figure 6b), has further been used to show that the mitochondrion of *P. falciparum* is able to assess Ca^2+^ fluctuations within the cell and can assist in the clearance of the divalent cation from the parasite cytoplasm by reversible accumulation.^[93]^ Many research efforts have been invested into ion dye development, with ongoing research to tune the colour of these sensors, allow ratiometric versus intensiometric (Figure 6c, d) imaging and to boost quantum yield and brightness.[^94^, ^95^]

With the established sensing of divalent Ca^2+^ ions, sensing of other physiologically relevant ions, such as Na^+^, K^+^, and Cl^−^, remains difficult due to the lack of selective ion binding scaffolds that can be synthesized, fused to a fluorophore and delivered into a living cell. Even pH sensors that rely on the concentration change of H^+^ are not as advanced as Ca^2+^ reporters. For instance, the ratiometric SNARF‐1 dye (Figure 6e) has been used in malaria research for high throughput screening of infected RBCs^[96]^ and intracellular pH fluctuations.^[97]^ It would indeed be interesting to use targeting probes, such as the above‐discussed Hoechst and various trackers, to fuse them to a Ca^2+^ and/or pH reporting fluorophore. Such a probe could be used in live cell imaging to observe changes in ion concentrations in distinct compartments in different infection stages.

In line with this, other molecular sensors have been reported, for example, for redox and signalling molecules H_2_S, glutathione and reactive oxygen species (ROS) and the reader is referred to these excellent reviews.[^98^, ^99^, ^100^] These species remain understudied in the context of examining *P. falciparum* and the toolbox of small molecular sensors available offers great potential.

### 3.4 Click chemistry and (photo)affinity labelling

**Figure 7:**
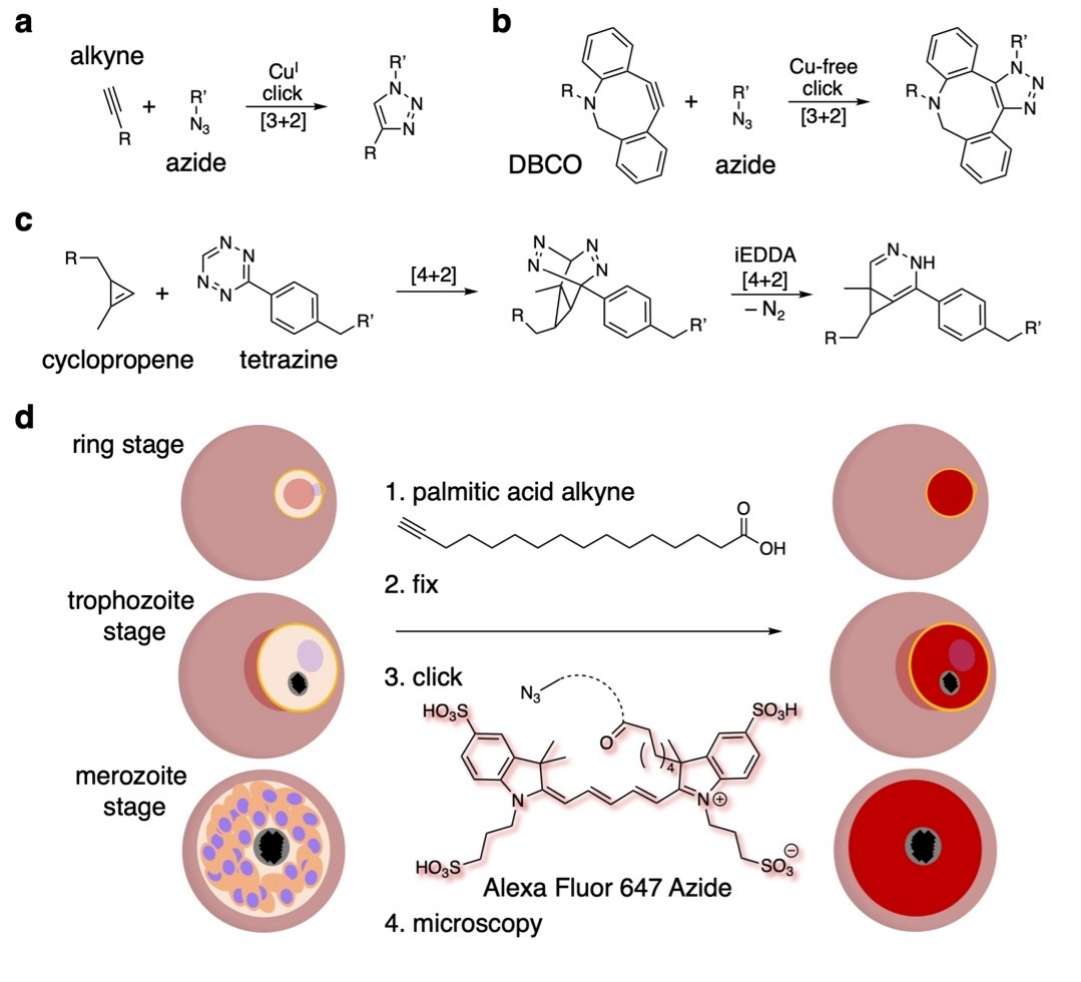
Click chemistry to stain the stages of *P. falciparum*‐infected red blood cells. a) An alkyne–azide click is catalysed by (potentially toxic) Cu^I^. b) Transition metal‐free, strain‐promoted alkyne–azide click chemistry using a dibenzocyclooctyne (DBCO). c) Cyclopropene tetrazine reactions undergo a [4+2] Diels–Alder reaction, and the resulting bicycle collapses in a retro‐[4+2] iEDDA reaction with extrusion of molecular nitrogen to yield a pyridazine. d) Membranes in the ring, trophozoite and merozoite stages of *P. falciparum*‐infected RBCs can be stained for microscopy by using a terminal‐alkyne‐bearing palmitic acid derivative, which, after fixation, can be clicked to Alexa Fluor 647 azide to allow the visualization of incorporated fatty acids.

Click chemistry is defined as a biorthogonal chemical reaction that takes place at physiological conditions, that is, at room temperature and in aqueous environments with quantitative outcomes,^[101]^ and has been used to investigate *P. falciparum* in recent years.[^4^, ^102^] Copper(I)‐catalysed alkyne azide click chemistry is probably the most famous out of a current large toolbox of different reaction modes^[103]^ (Figure 7a). Its reliance on toxic copper(I) limits the usage of this method to fixed cell applications, however, recently described developments circumvent the usage of transition metals.^[104]^ For instance, strain promoted alkyne azide click chemistry introduces strained triple bonds like in dibenzocyclooctyne (DBCO) to undergo an uncatalysed [3+2] cycloaddition with azides (Figure 7b). Tetrazines and cyclopropenes are another set of click partners where tetrazines need to be balanced between good stability and maintaining high and fast reactivity with the strained double bond of a cyclopropene in a cellular setting^[105]^ (Figure 7c). This reaction relies on an initial [4+2] Diels‐Alder reaction to form a bicycle, which continues to react in a reverse‐[4+2] inverse electron demand Diels‐Alder (iEDDA) reaction to finalize the click reaction.

An excess of mammalian cell applications could readily be listed; however, only a few have been used in parasitology research, even less so for imaging approaches. For instance, Mishra et al. reported on triazolyl glycoconjugates of cinchonidine in 2020, where they built a small library of 10 compounds by Cu^I^ catalysed click chemistry^[106]^ employing docking pointed towards plasmepsin inhibition of their novel molecules. Importantly, this study highlights the power of organic synthesis in the development of binders in a quick manner. Kilian et al. employed click chemistry in another study to simplify and accelerate the microscopic visualization and to investigate the behaviour of palmitoylated proteins in the different blood stages of *P. falciparum*.^[4]^ This was achieved by incorporating an alkyne into the palmitoyl chain, which is bioorthogonal, and allows a subsequent “click” to azide‐bearing fluorophores (i. e., Alexa647) after fixation (Figure 7d).

**Figure 8:**
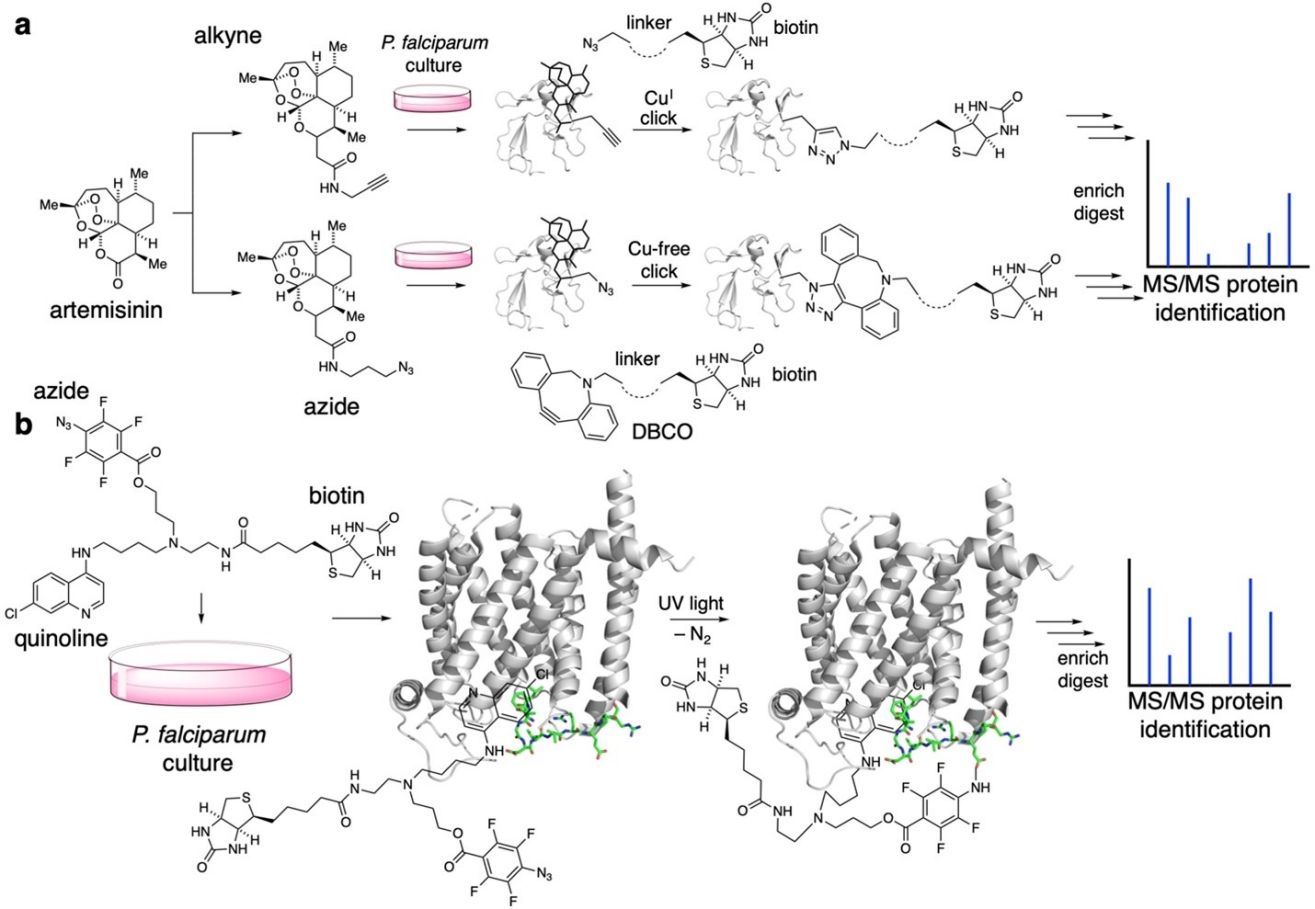
Click chemistry and affinity labelling. a) The antimalaria drug artemisinin can be derivatized to bear an alkyne (upper part) or an azide (lower part). Upon addition to *P. falciparum* culture, the artemisinin congener binds to its protein target merozoite surface protein‐1 (MSP‐1, PDB ID: 1N1I^[173]^), exposing the click handle, which can be reacted with a biotin‐bearing group. An alkyne–azide click can be performed using catalytic amounts of Cu^I^ (upper part) or using a strained dibenzocyclooctyne (DBCO, lower part) without the need for transition metals. The protein can now be enriched by biotin affinity, digested and characterized by mass spectrometry. b) A probe containing chloroquine, an azide and a biotin is added to *P. falciparum* culture, with the quinoline targeting the chloroquine resistance transporter (CRT, PDB ID: 6UKJ^[174]^). Upon exposure to UV light, the azide reacts with the protein in its vicinity (highlighted in green), creating a covalent linkage. The installed biotin handle can be enriched, the isolated protein can be digested and the binding vicinity can be determined using mass spectrometry comparable to (a).

An illustrative example using click chemistry in a medical chemistry and proteomics context are the studies by Ismail et al., who equipped the antimalaria drug artemisinin with an alkyne or an azide with almost no change in pharmacological binding and efficacy properties (Figure 8a). After binding of the artemisinin derivatives to proteins in *P. falciparum* culture, this probe allowed the click onto a biotin ligand. Depending on the click epitope, a protein bound alkyne was clicked to an azide with the use of Cu^I^, or on the other hand, was replaced by a strained dibenzocyclooctyne (DBCO) when using the azide‐artemisinin derivative. The biotin handle allowed subsequent enrichment and mass spectrometric analysis of bound proteins, which are abundant in “glycolytic, haemoglobin degradation, antioxidant defence and protein synthesis pathways”. The study concludes that artemisinin action shows a pleiotropic nature of biological function^[107]^ and is furthermore an example for affinity labelling strategies. While herein the labelling relies on strong, yet non‐covalent binding between the protein and the pharmacophore to ensure that the interaction will last during enrichment, covalent binders in affinity labelling circumvent this issue.

Affinity labelling describes the covalent linkage towards desired targets by equipping selective binders with a reactive moiety,^[108]^ for instance by linking a reactive electrophile (an NHS (*N*‐hydroxysuccinimide) or PFP (pentafluorophonol) ester) to a selective and high affinity pharmacophore. Once the pharmacophore is non‐covalently bound to a protein target, the active ester may react with a nearby nucleophile, such as a lysine residue, to form a covalent amide bond. Due to inherent disadvantages of activated esters, such as unspecific reaction and background hydrolysis, alternative methods were taken into consideration. Ultimately, light has been employed to trigger the covalent binding to add an external layer of control.[^109^, ^110^] As such, photolabile groups need introducing to pharmacophores, which are activated by a flash of UV light, generating highly reactive groups that link the molecule to the nearby protein surface. Additionally, other chemical groups can be introduced by this technique, for instance biotin, which exhibits strong binding affinity to streptavidin/neutravidin for later pulldown and enrichment. This approach elegantly offers the spatiotemporal control of illumination to be used in a cellular setting. In 2008, the Roepe group developed such a probe by considering three chemical parts: a chloroquine as the binding motif towards the chloroquine resistance transporter (CRT), an aryl azide photolabile group for crosslinking and a biotin moiety for enrichment^[111]^ (Figure 8b). After exposing their ligand to a culture of *P. falciparum*, where UV light triggered photoaffinity labelling, enrichment and mass spectrometry, the authors were able to determine the vicinity of the chloroquine binding site on the transporter, showcasing the power of custom‐tailored chemical biology probes. A similar study was reported in 2014 and used an azide‐functionalized albitiazolium, which is both photoactivatable and clickable on the same molecule.^[112]^ This allowed to first covalently label the target and then enrich by click chemistry for mass proteomic analysis and revealed that the drug mainly accumulated in the ER and Golgi and had effects on lipid metabolism.

In general, the binding motif is usually derived from pharmacophores, while the photoreactive group can furthermore be a benzophenone or a diazirine. While the former relies on a diradical mechanism for labelling after illumination with UV light, diazirines undergo nitrogen release and formation of a highly reactive carbene (similar to the azide used above), which may insert into nearby bonds.^[113]^ Berman et al. successfully applied photoaffinity labelling already in 1994 to assess proteins that are involved in phospholipid transport of older developmental blood stages of *P. falciparum* ^[114]^ and Foley et al. used the method to label proteins that bind the antimalarial drug chloroquine in 1993.^[115]^ Another more recently published manuscript focused on how PfMDR1 is able to interact with selected antimalarial drugs.^[116]^ While many advantages exist, photoaffinity labelling also exhibits some drawbacks. Illumination is usually performed with UV light, which can lead to cell damage and toxicity, especially when high intensity and long irradiation times are applied. Unwanted side reactions of unspecifically bound probes may complicate interpretation of the findings, and therefore the choice of the pharmacophore is of high importance. These factors have to be kept in mind in the development and use of photoaffinity labels.

### 3.5 Photopharmacology

**Figure 9:**
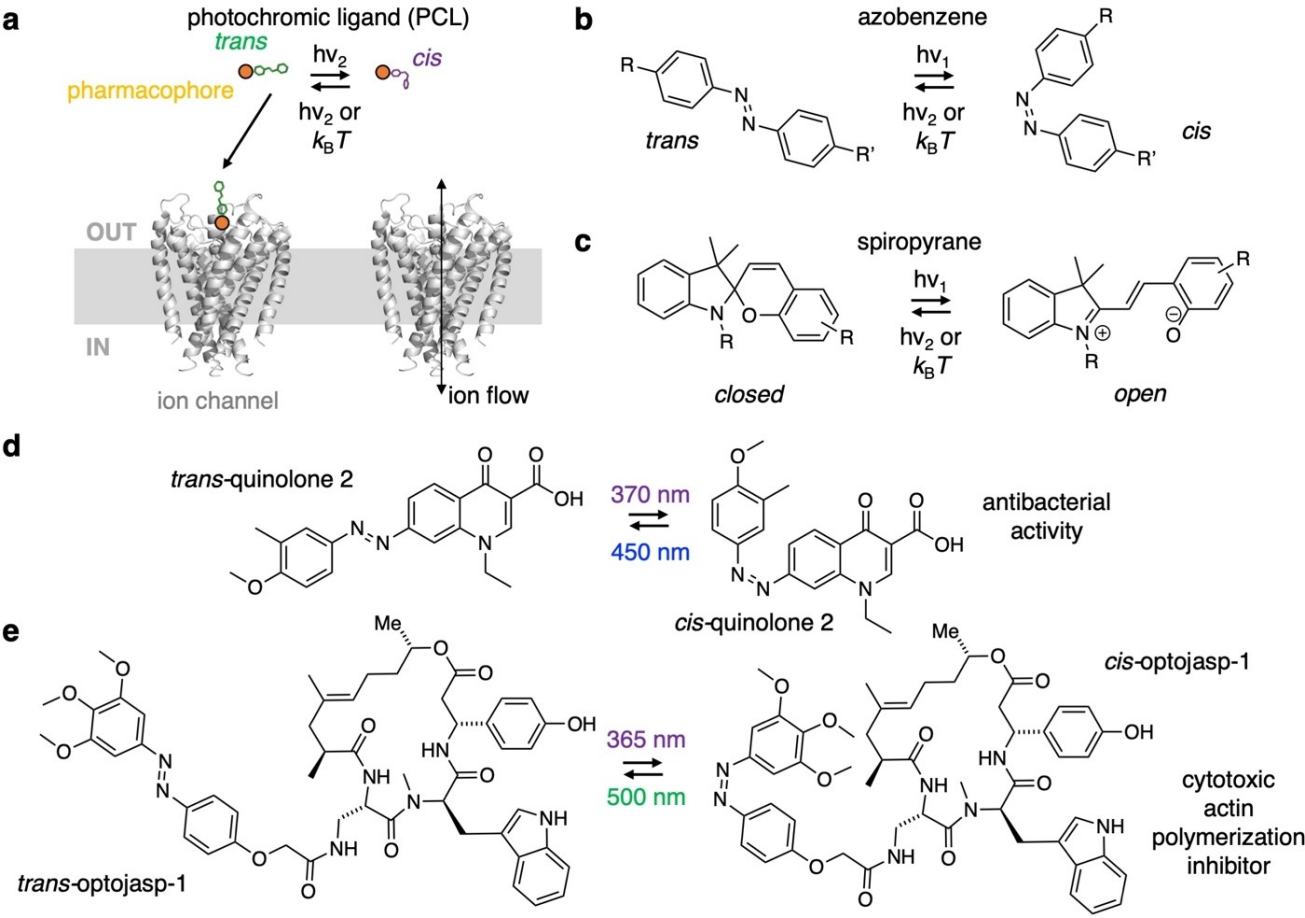
Photopharmacological concept and probes. a) Schematic representation of the optical control of ion channels (here bacterial potassium channel KCSA, PDB ID: 1 J95^[175]^). A pharmacophore (orange) is fused to a photochromic unit that can be toggled between two states by two orthogonal wavelengths of light or by thermal relaxation to the more stable isomer. While one isomer is able to bind and block the channel, the other is not, thereby allowing the flow of ion current. b) Azobenzenes and c) spiropyrans as examples for photoswitches that can be converted between their *cis*‐/*trans*‐ and open/closed states, respectively, by two orthogonal wavelengths or by thermal relaxation. The change in geometry and polarity affects the efficacy and efficiency of a fused pharmacophore (R and R’). d) A quinolone‐containing azobenzene exhibits antibacterial activity against *E. coli* CS1562 in its *cis* state (370 nm), while it remains pharmacologically silent in its *trans* state (450 nm). e) An azobenzene fused to a jasplakinolide derivative yields optojasp‐1, which inhibits actin polymerization in its *cis* form (365 nm), leading to cell death, while the *trans* state (500 nm) shows no pharmacological effect (*cf*. Figure 2).

Photopharmacology describes the use of light to control biological functions via small‐molecule photoswitches[^117^, ^118^, ^119^] (Figure 9). This approach favourably adds to the field of optogenetics, where light‐sensitive protein actuators (e. g., ion channels like channelrhodopsin, halorhodopsin)^[120]^ and/or sensors (e. g., Ca^2+^ and glutamate sensing proteins like GCaMPs and GluSnFRs, respectively)^[121]^ are transfected and thereby introduced into the system that is studied. Endowing light sensitivity to native proteins, however, is not achievable with genetic techniques. Still, it has been demonstrated in many instances with chemical photoswitches. This has allowed optical control in endogenous systems, ranging from cell‐surface receptors to the cytoskeleton, with the advantage of the unmatched spatiotemporal precision that is offered by light. A hallmark example is the optical control over potassium channel activity with photochromic ligands (PCLs; Figure 9a) in 2004.^[122]^ PCLs consist of a photoswitchable unit, for instance an azobenzene^[117]^ or spiropyran,^[123]^ which are fused to a (selective) pharmacological binder (Figure 9b, c). By applying light of a certain wavelength, the molecule changes its shape (*cis*/*trans* isomerism for azobenzenes, open and closed ring forms for spiropyrans), with ramifications of binding strength and/or efficacy. Importantly, this switch can be reversed by a second, orthogonal wavelength or by thermal relaxation (depending on the nature of the photoswitch) where the molecule adopts its thermodynamically most stable form. One prominent example is the use of a quinolone, which has been incorporated in a photoswitchable unit and is proposed to be used against bacterial infections,^[124]^ as it shows antibacterial activity in its *cis* state (Figure 9d). This could be an alternative to overcome antibacterial resistances. In another setting, actin inhibitor jasplakinolide can not only be fused to SiR (making SiR‐actin) for imaging, but also to an azobenzene to light control polymerization (Figure 9e).^[125]^ As the *cis*‐state of optojasp‐1 serves as the active blocker, it leads to cytotoxicity only in the illuminated area, highlighting the potential of its use among other probes in cancer therapy.[^126^, ^127^] With the advantage of no side effects in unilluminated regions, photopharmacology has, to the best of our knowledge, not yet been applied in parasitology research. It would be interesting if photopharmaceuticals, which are designed on demand and chemically synthesized accordingly, could be used to study *P. falciparum*. Given the broad applicability,^[128]^ it remains interesting to observe when photopharmacology will enter parasitology research.

### 3.6 Nanobodies

**Figure 10:**
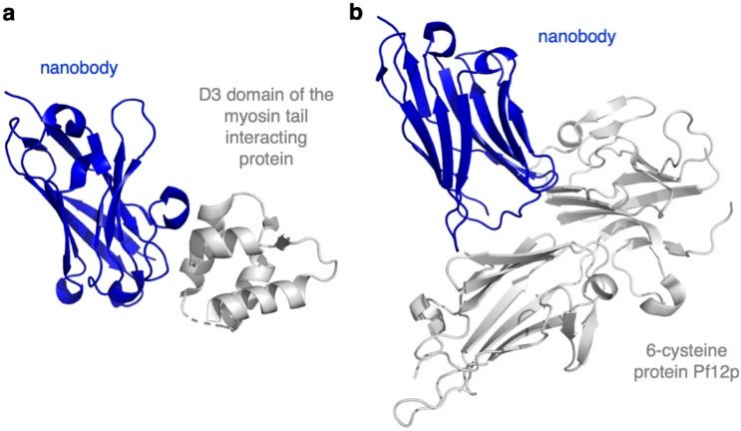
Nanobodies for *P. falciparum* protein crystallization. X‐ray structures were obtained for a) nanobody‐bound D3 domain of the myosin tail interacting protein (MTIP, PDB ID: 4GFT^[136]^), and b) the nanobody‐stabilized enigmatic 6‐cysteine protein Pf12p (PDB ID: 7KJI^[137]^).

Researchers have been on the lookout for strong and specific target binders for decades. Traditionally, antibodies are produced *via* the immunization of animals using peptide epitopes. Regarding small‐molecule binders, either high throughput screening has to be applied in conjunction with assay development or rationally designed probes need to be developed by tedious structure activity relationship screening, ideally based on crystal structures. While both approaches are powerful when finalized, they need excessive labour and time to be established. Nanobodies (or single‐domain antibodies (sdAb)) have squeezed into this design space. Based on single‐domain proteins, these proteogenic binders are usually derived from camelids or sharks and vary in size (∼15 kDa). Previously, nanobodies were also classically produced via animal immunization.^[129]^ Today, molecular biology approaches by means of ribosome and phage display are available to isolate many different nanobodies (termed sybodies) selective for a desired target from a DNA library or a bacteria library respectively within a few weeks.[^130^, ^131^] Similarly, yeast surface display can be used to raise nanobodies quickly for different protein conformers.^[132]^ Nanobodies can also be fused to fluorescent proteins to yield chromobodies,^[133]^ or to self‐labelling tags to deliver (photoswitchable) drugs.^[134]^ Nanobodies are a great alternative to antibodies and have already found their way into malaria research, for example as binders of non‐immunodominant domains of VAR2CSA, a PfEMP1 variant that facilitates the cytoadherence of the infected RBCs to CSA (chondroitin sulphate A) on syncytiotrophoblasts in the placenta.^[135]^ Lastly, nanobodies have received much attention for protein stabilization, in particular for structural elucidations using x‐ray crystallography and cryo‐electron microscopy. For instance, co‐crystals of nanobody bound structures of the D3 domain of the myosin tail interacting protein (MTIP)^[136]^ and 6‐cysteine protein 12p^[137]^ have been obtained by x‐ray diffraction (Figure 10). While MTIP is crucial for the malaria parasite to invade RBCs, 12p has been implicated with many functions ranging from transmission, host cell infection as well as reorganization and immune evasion. As we can learn much on the molecular level from detailed coordinates, it remains thrilling to see which sophisticated protein structures will emerge in the future.

### 3.7 Drug‐based probes

Probes based on (potential) antimalarial drugs bear great potential to investigate the subcellular localization of the compound within the *P. falciparum*‐infected erythrocyte while helping to decipher the mode of action.

Before synthetic efforts for new probes are started, some design criteria have to be considered. Especially for fluorescent imaging, a suitable fluorophore has to be chosen by answering important questions, that is, is fluorogenicity needed, and/or is a particular microscopic setup envisioned? If it needs to be chemically fused to a targeting moiety, that is, for a protein or an organelle, one has to decide on which linker (e. g., nonpolar lipid chains or polar and more soluble glycol chains) is appropriate and which chemistry should be used (e. g., amide bond coupling, click chemistry). If a particular biomolecular entity is within the scope, structure activity relationships of selective and strong binders help tremendously to choose an attachment site. Ideally, this is supported by crystal or cryo‐electron microscopy structures. Given that many fluorophores are commercially available, with congeners bearing clickable moieties (e. g., alkyne or azides), reactive amines or activated NHS ester, it makes sense to think about the targeting probe with a suitable handle so trackers can be installed late‐stage. To date, a good strategy is supported with empiric trials to determine suitable chemical biology probes that allow the investigation of parasites. Drug‐based probes in conjunction with click chemistry have been used for proteomics and library synthesis (*vide supra*). In 2017, the laboratory of Egan introduced NBD‐linked (nitrobenzoxadiazole) quinine and quinidine probes to visualize the blood stages of *P. falciparum* via confocal microscopy and super‐resolution structured illumination microscopy (SR‐SIM).^[138]^ Both NBD‐linked quinine and quinidine are very intriguing probes because they allow an almost immediate investigation of the parasite with negligible incubation time before the compound can be localized at the target compartment, while standard drug assays can take more time. Indeed, in a later study, the authors conclude that quinolines bind prominently to *P. falciparum* multidrug resistance‐associated protein (PfMRP1), modulating their activity.^[139]^ A 2018 published manuscript showed the localization of a fluorescent probe linked to the antimalarial drug artesunate (artesunate‐red), a semisynthetic derivative of artemisinin, within the mitochondria of HeLa cells.^[140]^ To our knowledge, however, this probe has not yet been applied for *P. falciparum*‐infected RBCs, but it verifies previously made antibody‐based observations concerning the subcellular localization of artemisinin within the parasite.^[141]^

**Figure 11:**
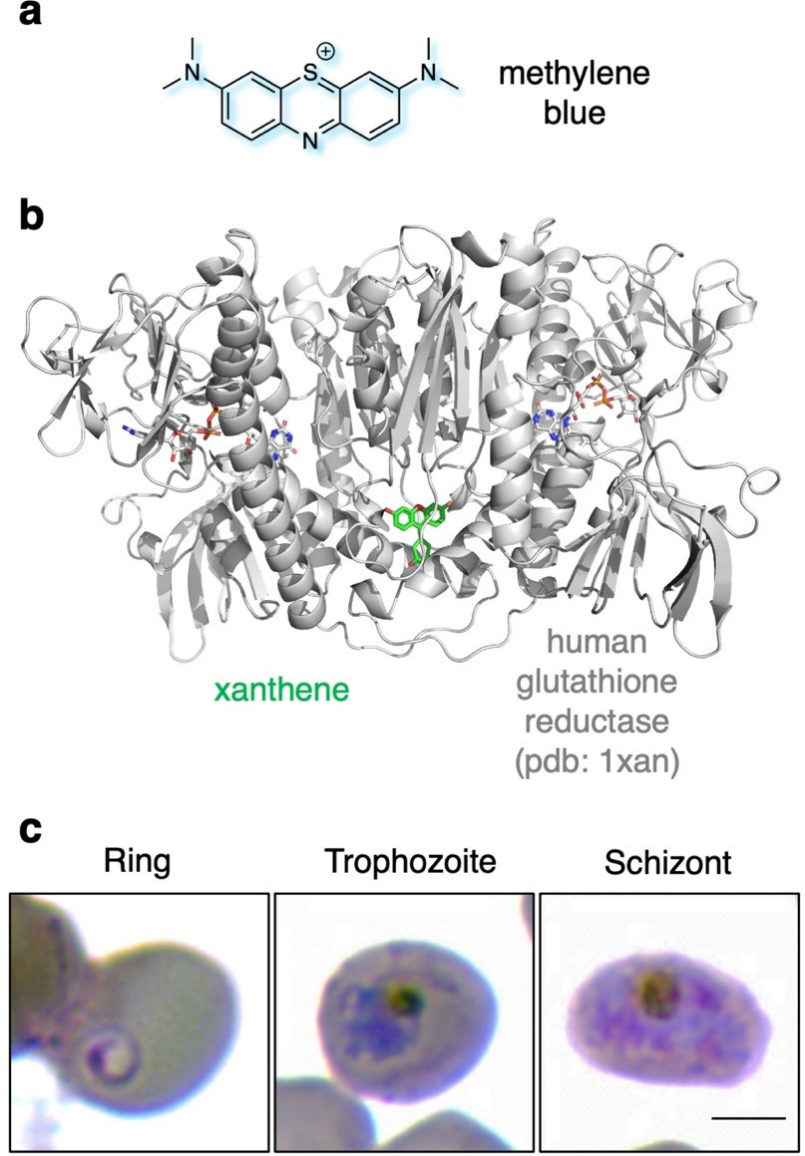
Methylene blue. a) Molecular structure of the oldest malaria drug methylene blue. b) *P. falciparum* glutathione reductase (here human, PDB ID: 1XAN^[176]^) is one target of methylene blue. Interestingly, the shown structure is bound to a xanthene, which has a similar scaffold to methylene blue. c) Different developmental stages of *P. falciparum*‐infected RBCs are shown by a Hema 3™ stain, comparable to Wright‐Giemsa staining. Methylene blue as a mixture with eosin and Azure B is known as the Giemsa stain to visualize parasites in histology. Scale bar: 3 μm. Modified with permission from ref. [4]. Copyright: 2020, Wiley.

The intrinsic fluorescence of some antimalarial drugs is another advantageous feature which makes additional synthesis steps unnecessary and allows an almost immediate visualization of the compound after the infected RBCs have been treated. Recently, Korkor et al. applied antimalarial pyrido[1,2‐a]benzimidazoles (PBI) and investigated the localization of the compounds within *P. falciparum*.^[142]^ Another example can be made of one of the most fascinating antimalaria drugs, methylene blue (Figure 11a). Discovered by Caro in the late 19th century, it was used in malaria treatment by Ehrlich at the beginning of the 20th century.^[143]^ While its mode of action remains not fully understood, it is known to treat methemoglobinemia (which happens during malaria infection) and as such is a sensitizer in combination with chloroquine therapy,^[144]^ breaking up haemoglobin crystals and inhibiting *P. falciparum* glutathione reductase (PfGR;^[145]^ Figure 11b). Standing on the WHO Model List of Essential Medicines, and part of the traditional Giemsa stain (Figure 11c) to quickly detect parasitological invasion,^[146]^ it bears more remarkable properties. As a thiazine dye with a large extinction coefficient (*ϵ*=45 500 m ^−1^ cm^−1^) in the far‐red (*λ*_max_=668 nm), it exhibits intrinsic, yet weak fluorescence, with a quantum yield *Φ*=0.01 (*cf. ϵ*_650 nm_ (SiR)=100 000 m ^−1^ cm^−1^), *Φ* (SiR)=0.40). Indeed, this would serve as a fantastic chemical biology probe with inherent pharmacology and detecting capabilities. Unfortunately, its brightness is too low to be used effectively in fluorescent microscopy – one might say “yet”, given the fast developments in the field to date.

## 4 Summary and Outlook

Chemical biology is a research field that has immensely contributed to our understanding of many different biological processes in various model organisms.^[147]^

The protozoan malaria parasite *P. falciparum* is an important model organism whose erythrocytic schizogony can be studied *in vitro*. However, investigating the excessive asexual replication of the parasite within the RBCs of the human host can be challenging and time consuming. Chemical biology probes are an excellent addition to the already existing method repertoire to investigate the pathophysiology‐defining part of the parasite's life cycle. This investigation is important because *P. falciparum* still remains a huge burden in endemic areas.

However, *P. falciparum* is not alone and there are other human‐pathogenic *Plasmodium* species that need to be investigated. The biology of these human‐pathogenic *Plasmodium* species differs markedly from *P. falciparum*. This becomes apparent when looking at the time it takes for these parasites to complete the erythrocytic schizogony, how many daughter parasites are produced during the erythrocytic schizogony, which kind of RBC is infected and how the host RBC is reorganized.[^21^, ^148^, ^149^, ^150^, ^151^, ^152^, ^153^] For example, the investigation of the human‐pathogenic *Plasmodium* species *P. vivax* and *P. knowlesi* is more challenging than the examination of *P. falciparum*. A proper *in vitro* RBC culture of *P. knowlesi* has only been established recently[^154^, ^155^] while the *in vitro* culture of *P. vivax*, which replicates within human reticulocytes, still remains challenging.^[156]^ The fact that many parasitologists deal with a model organism that is difficult to culture demands fast‐acting probes that would still allow an immediate investigation of the parasite, for example during short‐term *in vitro* cultures.[^157^, ^158^] Even more, non‐human‐pathogenic *Plasmodium* species exist, which are also of interest and must be investigated. *P. berghei*, a parasite that infects certain rodents, is one of several other non‐human‐pathogenic *Plasmodium* model organisms investigated to gain more insight into the biology of *Plasmodium* parasites. Commercially available dyes such as the ER‐Tracker Red, DAPI and Hoechst have, for example, already been applied to investigate this parasite species.[^159^, ^160^, ^161^]

The development of chemical biology probes, with applications mentioned herein, relies on organic synthesis and the introduction of fluorescent dyes. Robust probes are in high demand and their design is crucial: usually using an established strong binder and a bright and stable fluorophore, these two moieties have to be linked. The nature of the linker and its length rely on empiric trials, as was reported for SiR‐probes that target tubulin^[59]^ as well as the protease BACE1 (β‐site amyloid precursor protein‐cleaving enzyme 1) that is involved in Alzheimer's disease.^[162]^ Predictive approaches, guided by *in silico* calculations similar to other themes,^[163]^ to accelerate such designs would be beneficial for the community. In addition, the use of now available modern fluorogenic dyes and their behaviour needs a thorough investigation on the biomolecular target.[^164^, ^165^, ^166^] The chemistry usually relies on amide bond formation or on click chemistry, which both offer straightforward and late‐stage access to the desired probes. As such, a late‐stage installation of various fluorophores is desired. While design and synthetic efforts are one part, the availability of chemical biology probes is a concern. Developers and researchers ideally provide their latest probes readily upon request. This means, on the other hand, that chemical biologists need to have a workforce and a pipeline to have enough supply for such a demand. To circumvent the additional problem of short supply, it would be ideal to have a vendor supplying chemicals for an affordable price, ideally if single use aliquots can be provided at shipping cost. Given the fast development in the field, we are looking forward to the connection of researchers within different disciplines when they combine their efforts to tackle questions arising in parasitological research with small molecules.

## Computational Methods

X‐ray structures were visualized with PyMOL (Palo Alto, CA, USA). Graphs were plotted with GraphPad Prism 8. Structures were drawn in ChemDraw 19.0 (Perkin Elmer).

## Conflict of interest

The authors declare no conflicts of interests.

## Biographical Information

*Johannes ‘JB’ Broichhagen uses chemistry to seek new imaging modalities for biological applications. He obtained a Diplom in chemistry from the University of Erlangen–Nuremberg in 2010 and a PhD in photopharmacology with Dirk Trauner at the Ludwig Maximilian University Munich in 2014. Next, he conducted postdoctoral work with Kai Johnsson, first at the École Polytechnique Fédérale de Lausanne, and later at the Max Planck Institute for Medical Research in Heidelberg to learn about biomolecule labelling. Since March 2020, he has been an independent Junior Group Leader at the Leibniz‐Forschungsinstitut für Molekulare Pharmakologie in Berlin*.

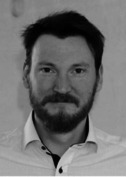

## Biographical Information

*After graduating in biology from Heidelberg University in 2008, Nicole Kilian pursued her PhD in the laboratory of Michael Lanzer at Heidelberg University Hospital, where she researched different aspects of the molecular mechanism underpinning protection from malaria by several structural hemoglobinopathies. She conducted postdoctoral research at Yale University into the Golgi apparatus in mammalian cells and photodamage during STED microscopy of mammalian cell lines, as well as protein trafficking and organelle establishment in Babesia‐infected red blood cells with James E. Rothman and Choukri Ben Mamoun, respectively. In 2019, she returned to Heidelberg to pursue her Habilitation in biology*.